# The association of marital status with kidney cancer surgery morbidity - a retrospective cohort study

**DOI:** 10.3389/fonc.2023.1254181

**Published:** 2023-10-02

**Authors:** Yuzhe Tang, Marie-Thérèse Valovska, José Ignacio Nolazco, Kendrick Yim, Benjamin Inbeh Chung, Steven Lee Chang

**Affiliations:** ^1^ Urology Department, Beijing Tsinghua Changgung Hospital, School of Clinical Medicine, Tsinghua University, Beijing, China; ^2^ Brigham and Women’s Hospital, Harvard Medical School, Boston, MA, United States; ^3^ Servicio de Urología, Hospital Universitario Austral, Universidad Austral, Pilar, Argentina; ^4^ Department of Urology, Stanford University Medical Center, Stanford, CA, United States

**Keywords:** marital status, kidney cancer surgery, surgical morbidity, retrospective cohort study, social support

## Abstract

**Purpose:**

To better understand whether the marital status impacts 90-day postoperative outcomes following kidney cancer surgery.

**Methods:**

We performed a retrospective cohort study of adult patients undergoing elective partial or radical nephrectomy to manage kidney masses from 2003 to 2017 using the Premier Hospital Database, a national hospital discharge dataset. Multinomial logistic regression models controlling for a wide range of clinicodemographic, surgical, and hospital characteristics were used to assess an association between marital status and postoperative complications. The primary outcome was 90-day complications, including minor complications (Clavien grades 1-2), non-fatal major complications (Clavien grades 3-4), and mortality (Clavien grade 5). Secondary outcomes included patient disposition and readmission rates.

**Results:**

The study cohort comprised 106,752 patients, of which 61,188 (57.32%) were married. The overall incidence of minor complications, major complications, and death was 24.04%, 6.00%, and 0.71%, respectively. Marriage was associated with a significantly lower incidence of minor (RR 0.97; 95% CI: 0.94-0.99) complications following open or radical nephrectomy and major complications (RR 0.89; 95% CI: 0.84-0.95) for all surgical types and approaches. There was no association between marital status and mortality (RR 0.94; 95% CI: 0.81-1.10).

**Conclusion:**

Marriage is associated with a significant reduction in major complications following kidney cancer surgery, likely because it is associated with greater social support, which is beneficial in the postoperative phase of care. Marital status and social support may play a role in the preoperative decision-making process and counseling for patients considering kidney cancer surgery.

## Introduction

Surgical outcomes are influenced by a complex interplay of factors, some more obvious than others. Recent studies have suggested that social factors such as socioeconomic status, social support, and living arrangement play an essential role in surgical outcomes. Social support encompasses a variety of factors, including the degree of social contact, the strength of relationships, faith, and marital status (1). Marital status, in particular, has been shown to have a substantial protective effect on survival and event-free rate in relative cancer survival for certain less lethal cancers (2) and specific postoperative patients – including those undergoing coronary revascularization and colorectal surgery (3, 4).

Prior studies suggest that this same benefit of marriage on outcomes also among patients with kidney cancer. For example, one study by Li et al. of almost 100,000 patients with renal cell carcinoma (RCC) demonstrated that married patients consistently had better 5-year overall survival (OS) and cancer-specific survival (CSS) than unmarried patients, including those who were single, divorced/separated, and widowed. Furthermore, the effect was persistent even after adjusting for sex, age, ethnicity, tumor grade, insurance status, histological type, stage, and therapy methods (5). Similar findings were demonstrated by Marchioni et al., who found that male, widowed, and separated/divorced patients had worse cancer control outcomes after treatment for stage T1-2 N0 M0 RCC (6). However, whether marital status offers this clinical benefit, specifically during kidney cancer surgery, remains unknown. Therefore, we used an extensive, nationally representative database to test the hypothesis that marital status improves outcomes after kidney cancer surgery to understand this potential relationship better.

## Methods

### Data source and study cohort

**Figure 1:**
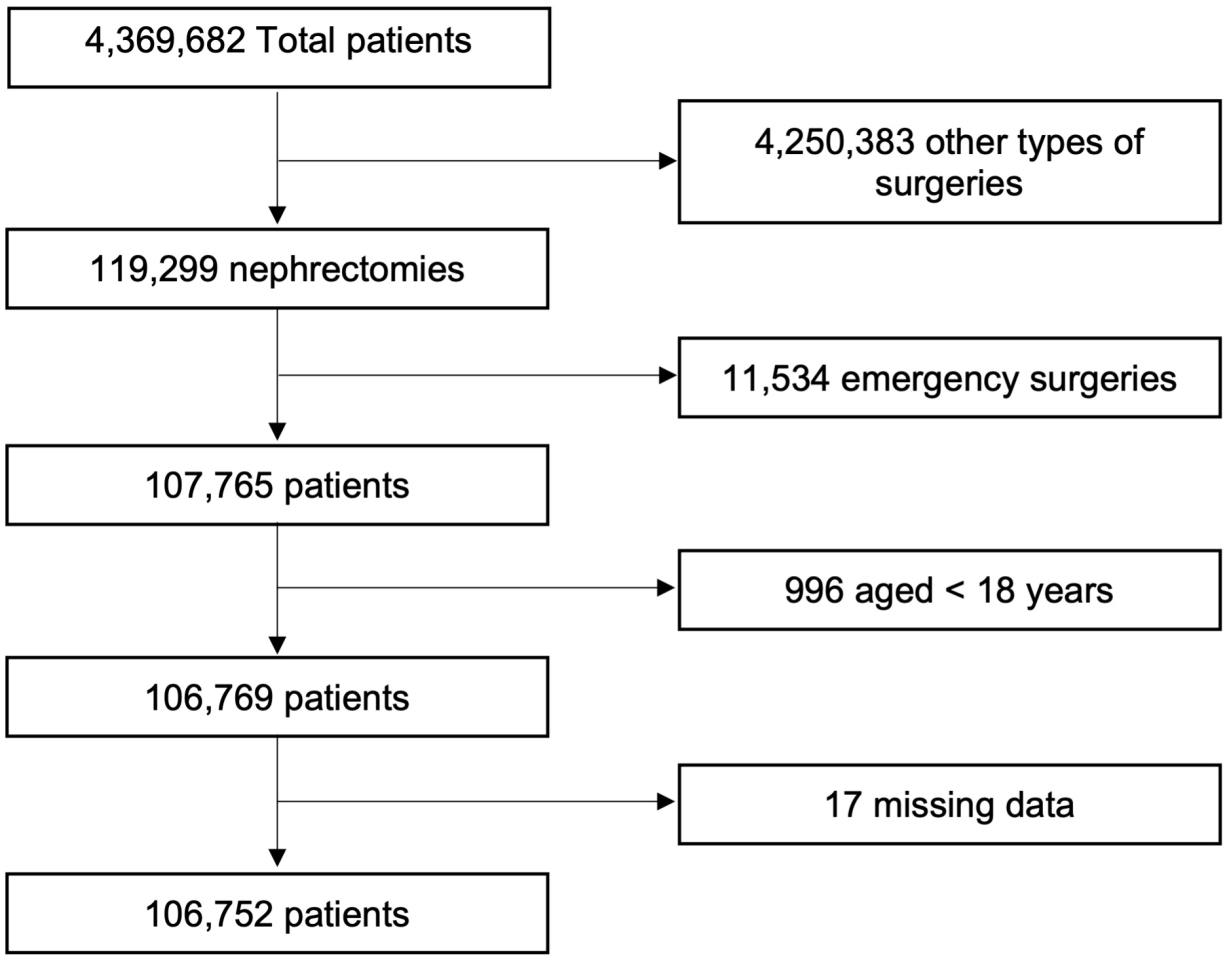
Consort diagram.

We conducted a retrospective cohort study using the Premier Healthcare Database (PHD, Premier Inc., Charlotte, NC), an extensive, US hospital-based, all-payer database representing approximately 20% of annual United States inpatient discharges at community and academic centers (7). The International Classification of Diseases, ninth revision (ICD-9), and tenth revision (ICD-10) procedure codes were used to identify patients who had undergone elective kidney cancer surgery between the 15 years of study from 2003 to 2017 (**Supplemental Table 1**). The cohort was then restricted based on appropriate ICD-9 and ICD-10 diagnosis codes to ensure that surgery was performed for a kidney mass (**Supplemental Table 1**). The study cohort was also limited to adult patients (age ≥18 years), “elective” cases based on administrative codes, as well as surgery on hospital day zero or one to minimize outlier patients who could skew the surgical outcomes (**Figure 1**).

### Demographic and clinical characteristics of the study cohort

We extracted demographic information from administrative data included in the PHD including age, gender, race, marital status, and insurance status. Clinical information was derived from ICD-9 and ICD-10 codes and subsequently used to calculate the Charlson comorbidity index, as has been previously described (8), which was segregated into three categories: 1-2, 3-4, and 5 and greater. The surgical characteristics, including the surgical approach (open, laparoscopic, robotic) and operating time, were derived from a review of the Chargemaster data in the PHD. Finally, surgeon and hospital cases were separated into quartiles per year as a surrogate for surgeon experience and hospital volume, respectively.

### Outcomes

The primary outcome of this study was the 90-day complication rate. Complication rates were based on the Clavien-Dindo classification of surgical complications as previously described (9) and divided into four categories: no complications, minor complications (Clavien grades 1, 2), and non-fatal major complications (Clavien grades 3, 4), and mortality (Clavien grade 5). Clinical systems also categorized complications (bleeding, cardiac, endocrine, gastrointestinal, infection, neurology, pulmonary, renal, soft tissue, urologic, venous thromboembolism) using Health Care Cost and Utilization Project Clinical Classification Software Level II or III designations; of note, the category for “surgical” complications was excluded given that all complications captured in this analysis are considered surgical complications. Secondary outcomes included patient disposition, length of hospital stay, and readmission rate.

### Statistical analysis

Categorical data were compared using the Chi-squared test. Normally distributed continuous data between married and unmarried groups were compared using the t-test. Non-normally distributed continuous data were compared using the Mann-Whitney U test. Standardized difference was used to compare the mean of the two groups. For the primary categorical outcomes, the proportional odds assumption was violated, a multivariable multinomial logistic regression model was used to adjust for confounding variables chosen based on literature reports and stepwise variables selection. Furthermore, a multivariable ordinal logistic regression model incorporating partial proportional odds was employed to address instances where certain variables exhibited violations of the proportion odds assumption. All clinicodemographic and surgical variables were included in the regression model, including age, gender, race, obesity, tobacco use, insurance payor, Charlson Comorbidity Index categories, operation time, surgery type (open vs. minimally invasive), nephrectomy type (radical vs. partial), surgeon volume categories (quartiles), hospital region, hospital type (academic vs. community), hospital volume categories (quartiles) and patients’ comorbidities (chronic obstructive pulmonary disease, myocardial infarction, congestive heart failure, hypertension, cerebrovascular disease, liver disease, chronic kidney disease, diabetes mellitus, peptic ulcer disease, hemiplegia, solid tumor). A multivariable logistic regression model was used to adjust the same variables for the secondary outcomes. Effect modification between age and marital status effect on patient disposition was also assessed. A multivariable linear regression model was used to adjust the same confounding variables for the continuous outcome, log-transformed length of stay. Due to the small percentage of missing data, we used the complete case analysis. Statistical analyses were conducted using Stata 17.0 (StataCorp LLC, College Station, TX, USA). The “gologit2” package was used in Stata for the partial proportional odds model. All p-values were reported as two-sided, and statistical significance was set to p<0.05. This work has been reported in line with the STROCSS criteria (10).

## Results

We identified 119,299 patients who underwent partial or radical nephrectomy between 2003 and 2017. A total of 12,547 patients were excluded based on exclusion criteria, leaving a cohort of 106,752 eligible patients, of which 61,188 (57.32%) were married. A greater proportion of married patients were male and Caucasian. Patient characteristics were shown in **Table 1**. Married patients also tended to have a lower incidence of comorbidities, including smoking status, diabetes, chronic kidney disease (CKD), congestive heart failure (CHF), and chronic obstructive pulmonary disease (COPD) but not hypertension or coronary artery disease (CAD). Rates of peripheral vascular disease were similar between groups.

**Table 1:** Baseline characteristics of patients, hospitals, and surgeons .

Factor	Unmarried	Married	Standardized Difference †
	45564(42.7%)	61188(57.3%)	
**Patients Characteristics**			
age, mean (SD)	61.4 (13.8)	62.0 (12.0)	0.04
Gender			0.32
Male	22646 (49.7%)	39949 (65.3%)	
Female	22918 (50.3%)	21239 (34.7%)	
Race			0.32
White	29505 (64.8%)	48388 (79.1%)	
Non-white	16059 (35.2%)	12800 (20.9%)	
Obesity			0.03
No	37873 (83.1%)	51516 (84.2%)	
Yes	7691 (16.9%)	9672 (15.8%)	
Urban or Rural			0.04
Rural	3127 (6.9%)	4863 (7.9%)	
Urban	42437 (93.1%)	56325 (92.1%)	
Smoking			0.08
No	29927 (65.7%)	42445 (69.4%)	
Yes	15637 (34.3%)	18743 (30.6%)	
Charlson Index Category			0.08
0-2	23686 (52.0%)	34168 (55.8%)	
3-4	13236 (29.0%)	16835 (27.5%)	
5+	8642 (19.0%)	10185 (16.6%)	
Hypertension			0.02
No	22532 (49.5%)	29527 (48.3%)	
Yes	23032 (50.5%)	31661 (51.7%)	
Diabetes			0.03
No	34505 (75.7%)	47072 (76.9%)	
Yes	11059 (24.3%)	14116 (23.1%)	
Chronic Kidney Disease			0.07
No	39267 (86.2%)	54170 (88.5%)	
Yes	6297 (13.8%)	7018 (11.5%)	
Coronary Artery Disease			0.02
No	39590 (86.9%)	52656 (86.1%)	
Yes	5974 (13.1%)	8532 (13.9%)	
Congestive Heart Failure			0.06
No	42784 (93.9%)	58267 (95.2%)	
Yes	2780 (6.1%)	2921 (4.8%)	
Chronic Obstructive Pulmonary Disease			0.10
No	37702 (82.7%)	52809 (86.3%)	
Yes	7862 (17.3%)	8379 (13.7%)	
Peripheral Vascular Disease			0.02
No	43578 (95.6%)	58767 (96.0%)	
Yes	1986 (4.4%)	2421 (4.0%)	
Metastasis			0.01
No	45078 (98.9%)	60473 (98.8%)	
Yes	486 (1.1%)	715 (1.2%)	
Insurance			0.14
Medicare	42771 (93.9%)	57324 (93.7%)	
Medicaid	2793 (6.1%)	3864 (6.3%)	
Managed Care	22178 (48.7%)	26592 (43.5%)	
Commercial Insurance	4464 (9.8%)	2122 (3.5%)	
Other Insurance	13115 (28.8%)	23846 (39.0%)	
**Hospital and Surgery Characteristics**	2988 (6.6%)	5456 (8.9%)	
Region	2819 (6.2%)	3172 (5.2%)	0.02
Midwest	8774 (19.3%)	11708 (19.1%)	
Northeast	9226 (20.2%)	10585 (17.3%)	
South	19689 (43.2%)	28805 (47.1%)	
West	7875 (17.3%)	10090 (16.5%)	
Hospital Beds Volume			0.05
< 200	4468 (9.8%)	6259 (10.2%)	
200-299	5970 (13.1%)	8384 (13.7%)	
300-399	7436 (16.3%)	11486 (18.8%)	
400-499	6797 (14.9%)	8610 (14.1%)	
≥ 500	20893 (45.9%)	26449 (43.2%)	
Teaching Hospital			0.06
No	20682 (45.4%)	29738 (48.6%)	
Yes	24882 (54.6%)	31450 (51.4%)	
Hospital Volume			0.02
Q1	11614 (25.5%)	14964 (24.5%)	
Q2	10857 (23.8%)	15620 (25.5%)	
Q3	10711 (23.5%)	15842 (25.9%)	
Q4	12382 (27.2%)	14762 (24.1%)	
Surgeon Volume			0.02
Q1	11792 (25.9%)	15570 (25.4%)	
Q2	10709 (23.5%)	15154 (24.8%)	
Q3	10647 (23.4%)	15424 (25.2%)	
Q4	12416 (27.2%)	15040 (24.6%)	
Operation Time(h), median (IQR)	3.8 (2.9, 5.0)	3.8 (2.9, 5.1)	0.01
Operation Type			0.09
Open	22178 (48.7%)	26592 (43.5%)	
Minimally Invasive	18946 (41.6%)	28021 (45.8%)	
Nephrectomy Type			0.05
Radical	30949 (67.9%)	40059 (65.5%)	
Partial	14615 (32.1%)	21129 (34.5%)	

† Standardized difference greater than 0.1 indicantes imbalance

Our results showed that married patients had a lower likelihood of experiencing both minor and major complications within the 90-day postoperative period, but mortality was similar between groups. Univariable analysis showed that the unadjusted Risk ratio (RR) of minor and non-fatal major complications was 0.95 (95%CI 0.92-0.98, p<0.01) and 0.83 (95%CI 0.79-0.88, p<0.01), respectively. The unadjusted mortality RR was 0.89 (95%CI 0.77-1.02, p=0.10). After accounting for potential confounding variables, the adjusted RR of minor and major complications was 0.97 (95%CI 0.94-0.99, p=0.04) and 0.89 (95%CI 0.84-0.95, p<0.01), respectively. The adjusted RR of mortality was 0.94 (95%CI 0.81-1.10, p=0.45). The RR and 95%CI based on the adjusted multinomial logistic regression models are illustrated in **Table 2**. The Odds Ratio (OR) from the partial proportional ordinal logistic regression model is 0.95 (95%CI 0.93-0.98, p<0.01)

**Table 2:** Adjusted RR and 95% CI for different complications.

	RR	95% CI	P value
Minor complications	0.97	0.94-0.99	0.04
Non-fetal major complications	0.90	0.84-0.95	<0.01
Mortality	0.94	0.81-1.10	0.45

**Figure 2:**
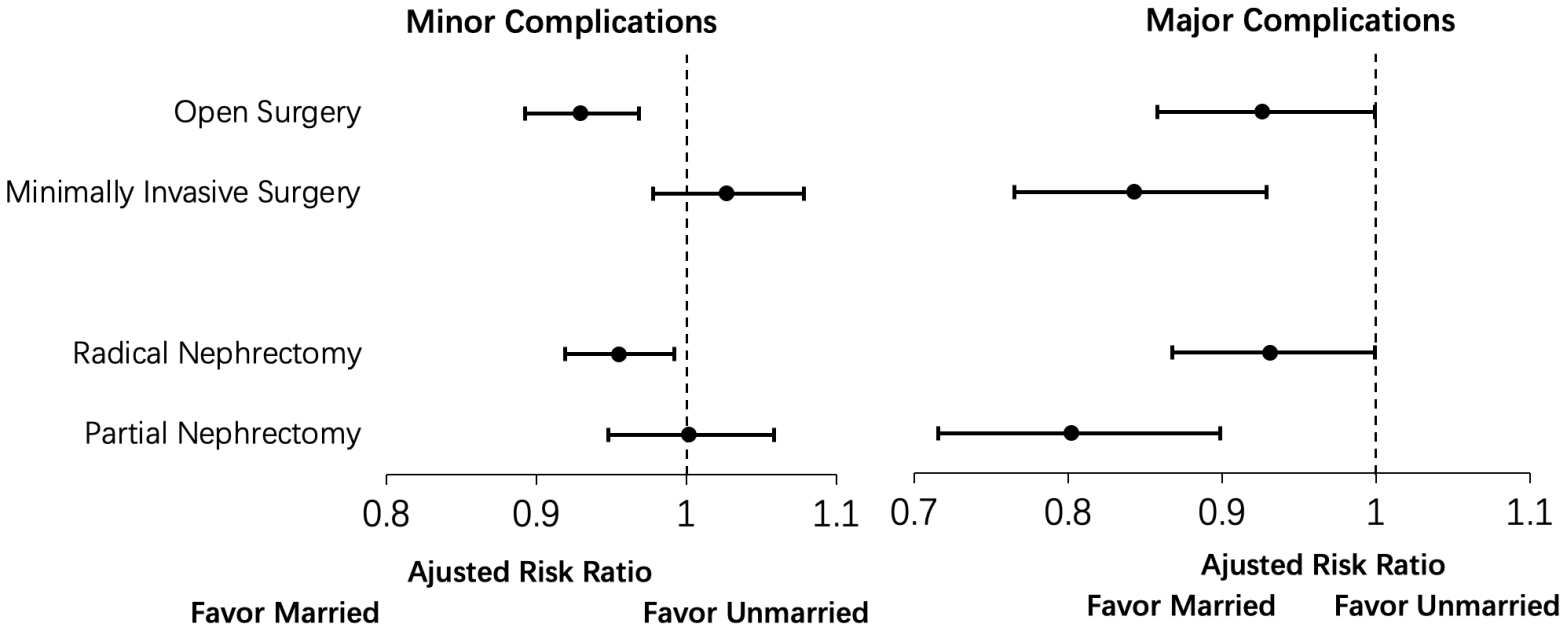
Subgroup analysis for surgical approach (open versus minimally invasive surgery) and type of surgery (radical nephrectomy versus partial nephrectomy) for the association of martial status with 90-day minor and major complications.

**Figure 3:**
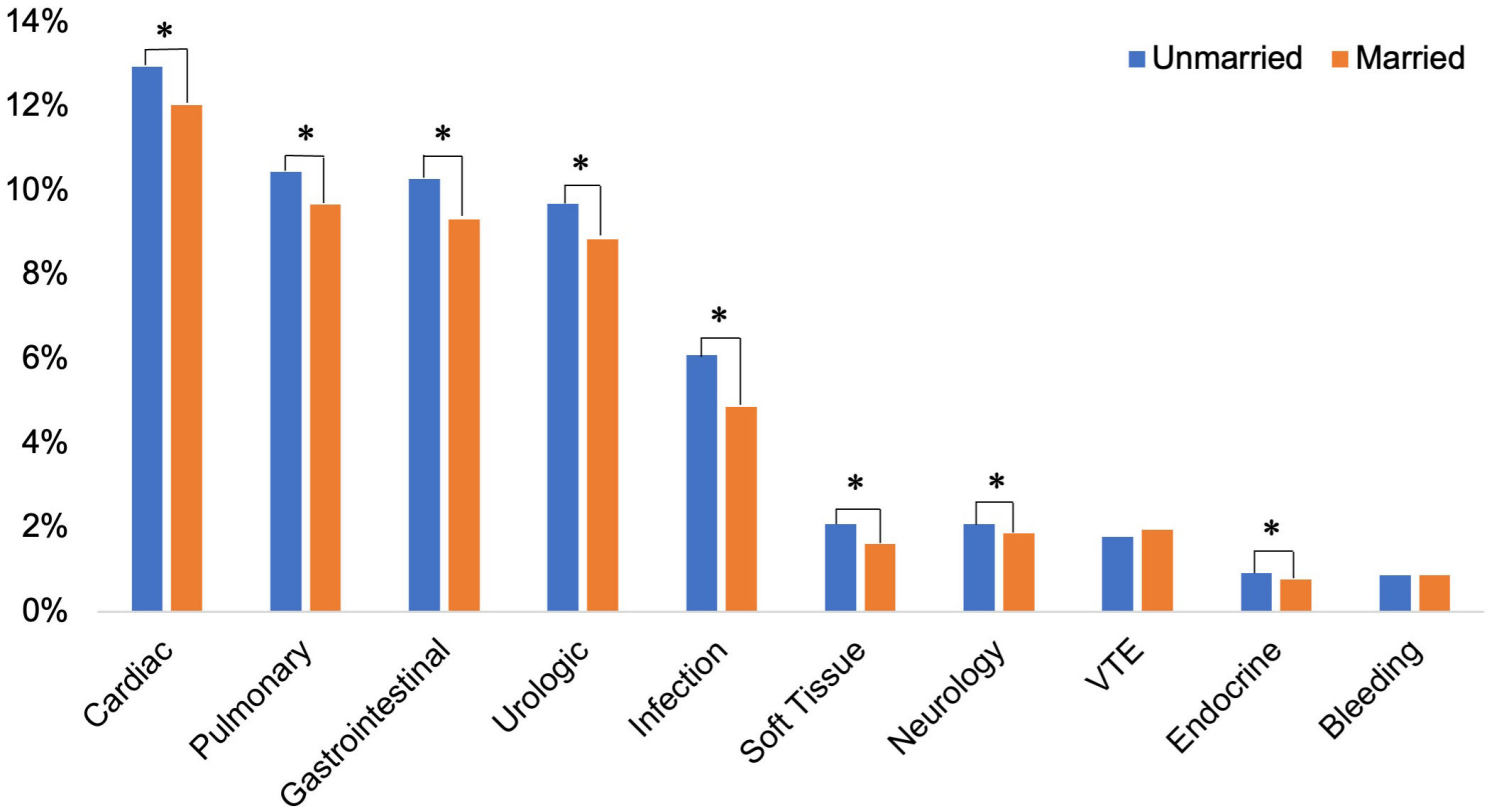
Analysis of postoperative complications categorized by clinical systems.

Multivariable multinomial logistic regression was also applied to subgroup analysis, which focused on the type of surgery (radical and partial), surgical approach (open and minimally invasive), and an evaluation of complications by the clinical system. Our analysis demonstrated that marriage was associated with fewer minor complications following open or radical nephrectomy. In comparison, marriage was associated with fewer major complications across all surgical and surgical approaches (**Figure 2**). Furthermore, when evaluating the specific type of complication by the clinical system, our model revealed that the predicted probability for complication rates was statistically significantly lower in the married group for all complication subtypes except for bleeding and venous thromboembolism (VTE) complications where there were no differences between married and unmarried patients (**Figure 3**).

**Figure 4:**
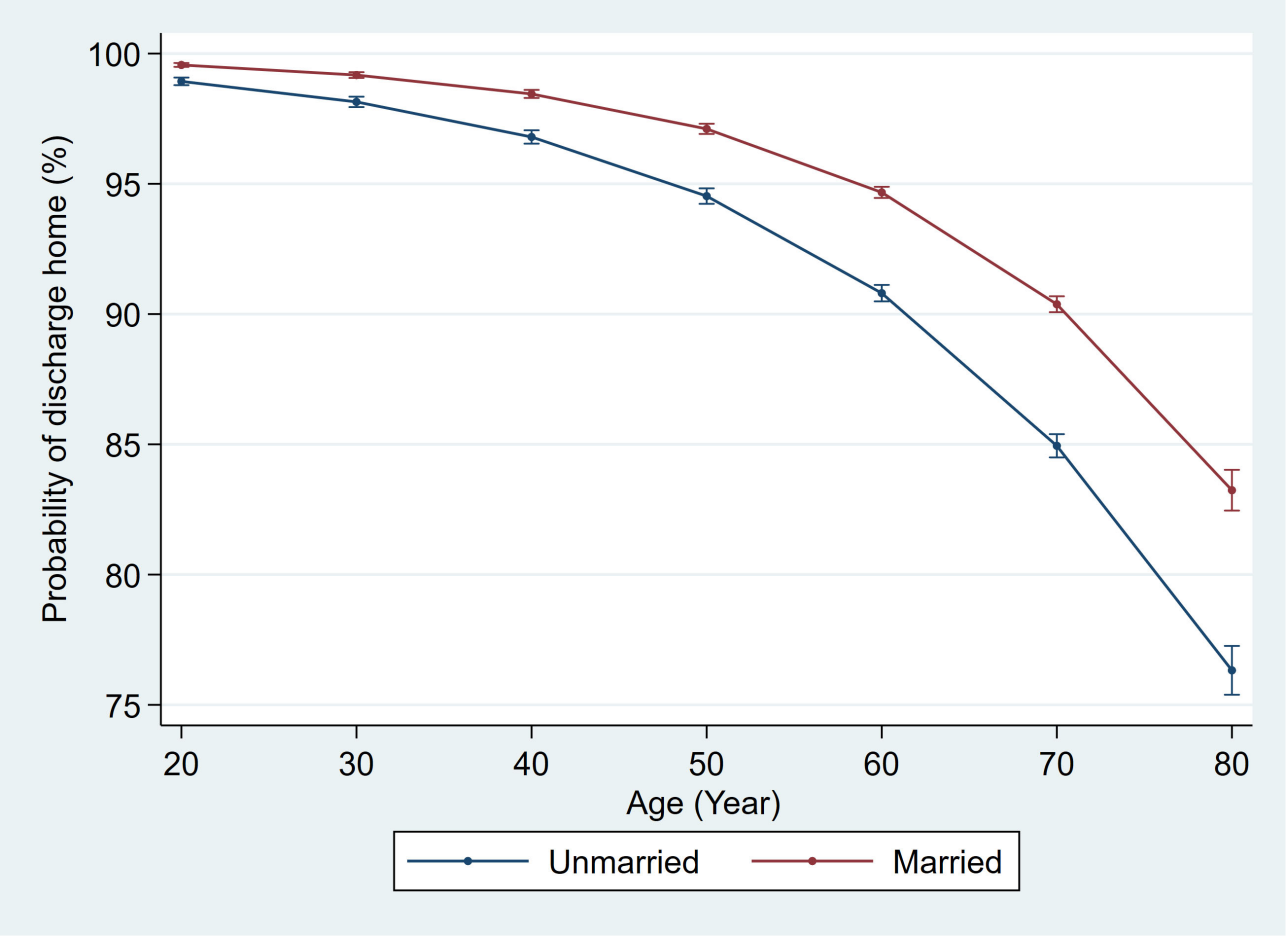
The effect of age on the marital status and postoperative discharge home.

Discharge disposition and readmission rate were also analyzed using the multivariable logistic regression model; the same variables were used to adjust for confounding. The model showed married patients were significantly more likely to be discharged home compared to unmarried patients (OR: 2.92 [95%CI 2.21-3.85, p<0.01]). This disparity in discharge disposition between married and unmarried patients increased with advancing age such that unmarried patients were significantly more likely to be discharged to a skilled nursing or rehabilitation facility rather than home (**Figure 4**). A statistically significant length of stay between married and unmarried patients was detected but not clinically meaningful (Ratio of mean length of stay 0.97 95%CI 0.96-0.97, p<0.01). There were no statistically significant differences between the readmission rate for the two groups (OR 1.00; 95%CI 0.97-1.03, p=0.99).

## Discussion

Our results showed that marriage is associated with approximately 3% lower risk of a minor complication and 10% lower risk of non-fatal major complications following kidney cancer surgery, but no appreciable difference in mortality. Furthermore, these differences persisted in subgroup analyses with respect to major complications but were attenuated for minimally invasive surgery and partial nephrectomy concerning minor complications. The results of the partial proportional odds model suggest that being married is associated with 5% lower odds of experiencing any complications. Our study suggests that social support especially from spouse likely plays an essential role in recovering from kidney cancer surgery. As such, marital status and other social support status should be incorporated into the preoperative decision-making process and counseling for patients undergoing kidney cancer surgery.

Although the association between marital status and improved surgical outcomes have been previously identified in several studies (5, 11), this is the first study documenting this relationship in a large cohort of patients undergoing kidney cancer surgery. However, our findings are consistent with other studies investigating this association in patients undergoing surgery for genitourinary malignancies. For example, Sammon et al. (2012) studied 14,859 patients who underwent radical cystectomy for urothelial carcinoma of the urinary bladder; they found that never-married males had a significantly higher rate of non-organ confined disease at cystectomy and that separated, divorced, or widowed males and females had significantly higher rates of bladder-cancer-specific mortality than married males and females (12). More recently, Ruvolo et al. (2021) identified 8833 non-metastatic upper tract urothelial carcinoma patients treated with radical nephroureterectomy. In this study, unmarried male and female patients had significantly higher overall mortality, though cancer-specific mortality was only significantly higher in unmarried male patients (13). Another SEER database based retrospective study also showed that in the renal clear cell carcinoma population, those who were married had a higher overall survival rate than those who were not married (14).

The protective effects of marriage can be explained by several mechanisms, all rooted in the fact that marriage can be a vital source of social support. First, spouses of married patients may encourage their partners to seek earlier care or pursue surgical treatment. Using surgery type as a surrogate of tumor stage, given that partial nephrectomies are typically performed for smaller kidney masses than radical nephrectomies, we found a slightly higher proportion of partial nephrectomy in married patients (34.6% vs. 32%, p<0.01), raising the possibility that married patients are diagnosed with kidney cancer at an earlier stage, or pursue surgical treatment more expeditiously. Secondly, spouses can provide physical care and emotional support pre-and post-operatively, accelerating recovery. Although parents, children and friends can also provide physical care and emotional support for unmarried people, the support and care from a spouse may be more lasting, especially for preoperative lifestyle improvements. Finally, the partners of patients can influence their health-related behaviors. Our cohort found that married patients were significantly less likely to smoke than married patients (30.5% vs. 34%, p<0.01) and were less likely to have several comorbidities, including diabetes, CKD, CHF, and COPD. Beyond optimizing the preoperative health status, spouses can also beneficially influence postoperative recovery with improved to adhere to post-surgical instructions and early detection of postoperative complications.

Though marital status was associated with a lower incidence of minor and major 90-day complications, there was no association between marital status and postoperative mortality. There are two possible explanations for this finding. First, while social support may be powerful enough to influence complication rates, its impact may not be powerful enough to prevent mortality. There may be too few deaths overall to discern a meaningful difference. Additional study will be necessary to understand better why marital status is unrelated to postoperative morality while there is a relationship with postoperative non-fatal morbidity.

Furthermore, we observed a consistent advantage among married patients to postoperative major complications among open and minimally invasive surgery and radical nephrectomy subgroups. Still, this difference was not uniform for minor complications (**Figure 2**). It is plausible that our data more accurately captures major complications that commonly require management within the hospital; in contrast, minor complications that can be addressed in the outpatient setting (e.g., oral antibiotics) may not be captured in our dataset. Thus, we would fail to appreciate any differences. A further study utilizing a database that comprehensively includes outpatient data will be necessary to explain better the absence of differences in postoperative minor complications for minimally invasive surgery and partial nephrectomy.

Our results also confirm that marriage could decrease the major complication rate but neither impact the readmission rate nor the length of stay. The main reason may be that marriage reduces the incidence of major complications, which are relatively low at 5.63%, unlike minor complications, and therefore do not affect the overall readmission rate or reduce the average length of stay for the overall population.

In the immediate postoperative period, married patients were significantly more likely to be discharged home than unmarried patients. This is because spouses play a vital role in postoperative patient care, and therefore a patient with support at home is more likely to be deemed safe for a home discharge. This disparity in postoperative disposition was more pronounced with increasing age. The absolute difference in probability of disposition home was 2.18% for 40-year-old patients, 4.49% for 60-year-old patients, and 6.67% for 80-year-old patients (**Figure 4**). This is not particularly surprising since the likelihood for comorbidities and frailty rises with age, thus raising the threshold for discharge home versus another care facility; it is among these most at-risk patients that having the support of a spouse likely has the most significant impact.

Overall, our study suggests that social support in the form of marriage plays a vital role in reducing 90-day postoperative complications in patients undergoing kidney cancer surgery. Therefore, marital status as a marker of a solid social support system should be considered pre-operatively when assessing the risk-to-benefit ratio for surgical intervention. In other words, surgeons should be aware that unmarried patients undergoing kidney cancer surgery may be more likely to require additional resources upon discharge, such as a visiting nurse association (VNA) or home physical therapy (PT). These considerations may influence, for instance, the decision for a technically challenging partial nephrectomy versus a radical nephrectomy when other clinical issues (e.g., risk of postoperative chronic kidney disease) are relatively equal between the two options. Of course, we recognize that marital status in this study is merely a potential proxy for a support system, and non-married patients may nevertheless have a robust social network. Clinicians must recognize that similar support could be derived from myriad other sources, which may vary on an individual basis.

There are, of course, several limitations to this study. First, this is a retrospective cohort study based on administrative data with inherent biases and a lack of control of unmeasured confounders; however, it is not feasible to conduct a prospective controlled trial for a demographic characteristic such as marital status. Second, the PHD dataset does not capture tumor characteristics, which precludes adjustment for tumor complexity in our analysis. However, our findings were consistent even in the subgroup analysis of partial nephrectomies, which likely have the homogeneously low stage and favorable tumor characteristics, suggesting that our conclusions were robust. Third, while there is no perfect measure of surgeon skill and hospital resources, we controlled the annual surgeon and hospital volume and overall hospital bed number to minimize unmeasured biases. Fourth, the marriage status in our study only captures the status of married vs. unmarried. There could be separated couples in the married group and divorced or widowed in the unmarried group, which might differ from the other people in the same category. We cannot distinguish between such groups, but social support in marriage groups is often attenuated for separated partners. In addition, social support from marriage is not available for widowed or divorced people. Hence, our result is a conservative estimate. Lastly, social support is a broad category that includes marriage and other factors, including family support, the strength of relationships, and the degree of social contact. Some married patients may have strained relationships, whereas other unmarried patients may have a robust social network. Because our study’s mean age is above sixty, the social support from marriage as an entity has evolved. Social support from other sources, such as adult children, may also play an important role. Nevertheless, our findings were consistent after adjusting for confounders and subgroup analyses, supporting that marital status is a reliable measure of social support, impacting the perioperative course for patients undergoing kidney cancer surgery.

## Conclusion

Marriage is associated with a lower incidence of minor and major 90-day complications in patients undergoing kidney cancer surgery. While marriage can be a vital source of social support, its impact may not be substantial enough to impact overall mortality. Marital status as a measure of general social support should be considered when assessing surgical risk-to-benefit ratios, and unmarried patients should be offered additional support in the perioperative period. Clinicians should continue to consider all potential sources of social support when evaluating a patient for surgery.

## Data availability statement

The original contributions presented in the study are included in the article/**Supplementary Material**. Further inquiries can be directed to the corresponding author.

## Ethics statement

The studies involving humans were approved by Brigham and Women’s Hospital Institional Review Board. The studies were conducted in accordance with the local legislation and institutional requirements. Written informed consent for participation was not required from the participants or the participants’ legal guardians/next of kin in accordance with the national legislation and institutional requirements. This retrospective study which presents no more than minimal risk of harm to subjects was approved by IRB, the informed consent was waived.

## Author contributions

YT: Conceptualization, Data curation, Formal Analysis, Investigation, Methodology, Project administration, Resources, Software, Validation, Visualization, Writing – original draft, Writing – review & editing. M-TV: Investigation, Visualization, Writing – review & editing. JN: Software, Validation, Visualization, Writing – review & editing. KY: Writing – review & editing. BC: Resources, Writing – review & editing. SC: Conceptualization, Data curation, Formal Analysis, Investigation, Methodology, Project administration, Resources, Software, Supervision, Validation, Visualization, Writing – review & editing.
