# Supplementary material for: The association of marital status with kidney cancer surgery morbidity - a retrospective cohort study
Source: Front Oncol. 2023 Oct 2;13:1254181. doi: 10.3389/fonc.2023.1254181 (PMC10577411; doi:10.3389/fonc.2023.1254181)
Supplement: Supplementary file 1 [file Table_1.docx]

|  | **ICD-9 codes** | **ICD-10 codes** |
| --- | --- | --- |
| **Procedure codes** | 55.51, 55.52, 55.53, 55.54 | 0TT00ZZ, 0TT10ZZ, 0TT04ZZ, 0TT14ZZ, 0TB00ZZ, 0TB10ZZ, 0TB03ZZ, 0TB13ZZ, 0TB04ZZ, 0TB14ZZ, 0TB07ZZ, 0TB17ZZ, 0TB08ZZ, 0TB18ZZ |
| **Diagnosis codes** | 223.0, 223.1, 189.0, 189.8, 189.9, 198.0, 198.1, 236.91, 239.5, 593.2, 593.9, 753.10, 753.11, 753.19 | C64.1, C64.2, C64.9, C79.0, N28.1, Q61.00, Q61.01, Q61.02, N28.89, D30.00, D30.01, D30.02, D41.00, D41.01, D41.02, D49.511, D49.512, D49.519, D49.59 |

**Supplementary table 1 :** ICD-9 and ICD-10 codes for extracting the kidney surgery cohort
